# Randomized study of temporary diaphragm pacing for enhanced recovery after surgery in cardiac surgery patients at risk of prolonged mechanical ventilation

**DOI:** 10.1016/j.xjon.2024.09.031

**Published:** 2024-10-18

**Authors:** Jessica R. Hungate, Raymond P. Onders, Mohammad El Diasty, Yasir Abu-Omar, Rakesh C. Arora, Cristian Baeza, Yakov Elgudin, Kelsey Gray, Alan Markowitz, Marc Pelletier, Igo B. Ribeiro, Pablo Ruda Vega, Gregory D. Rushing, Joseph F. Sabik

**Affiliations:** a Division of Cardiac Surgery, University Hospitals Cleveland Medical Center, Cleveland, Ohio; b Division of General and Gastrointestinal Surgery, Department of Surgery, University Hospitals Cleveland Medical Center, Cleveland, Ohio

**Keywords:** diaphragm pacing, Enhanced Recovery After Surgery, prolonged mechanical ventilation

## Abstract

**Objective:**

Prolonged mechanical ventilation after cardiac surgery significantly increases morbidity and mortality. The aim of this study is to establish the role of diaphragmatic pacing to decrease mechanical ventilation burden in high-risk patients undergoing cardiac surgery.

**Methods:**

This is a prospective, randomized trial of temporary diaphragmatic pacing electrode use in patients undergoing cardiac surgery (NCT04899856). Prognostic enrichment strategy was used to identify patients at higher risk of prolonged mechanical ventilation by having inclusion criteria of prior open cardiac surgery, left ventricular ejection fraction less than 30%, history of stroke, intra-aortic balloon pump, or history of chronic obstructive pulmonary disease. Two electrodes were placed in each hemidiaphragm intraoperatively. On arrival to the intensive care unit, patients were randomized to immediate diaphragmatic pacing or standard of care.

**Results:**

Forty patients received implants, with 19 in the treatment group and 21 in the standard of care group. Only 1 patient in the treatment group was on mechanical ventilation at 24 hours versus 4 patients in the standard of care group, resulting in a relative risk reduction of 71% being on mechanical ventilation at 24 hours postoperatively. Predictive enrichment strategy was used to identify patients most likely to respond to therapy of diaphragmatic pacing. In this analysis, median time on mechanical ventilation was 17.7 hours (interquartile range, 8.3-23.4) for the 15 patients in the standard of care group and 9.4 hours (interquartile range, 7.14-12.5) for the 13 patients in the treatment group, for an improvement of 8 hours with diaphragm pacing (*P* < .05).

**Conclusions:**

Temporary diaphragmatic pacing improved weaning from mechanical ventilation by 8 hours with a significant reduction of prolonged mechanical ventilation. Multicenter randomized trials confirming diaphragmatic pacing as an Enhanced Recovery After Surgery tool to decrease mechanical ventilation may reduce length of stay, postoperative infections, and additive costs.

## Graphical Abstract

**Figure:**
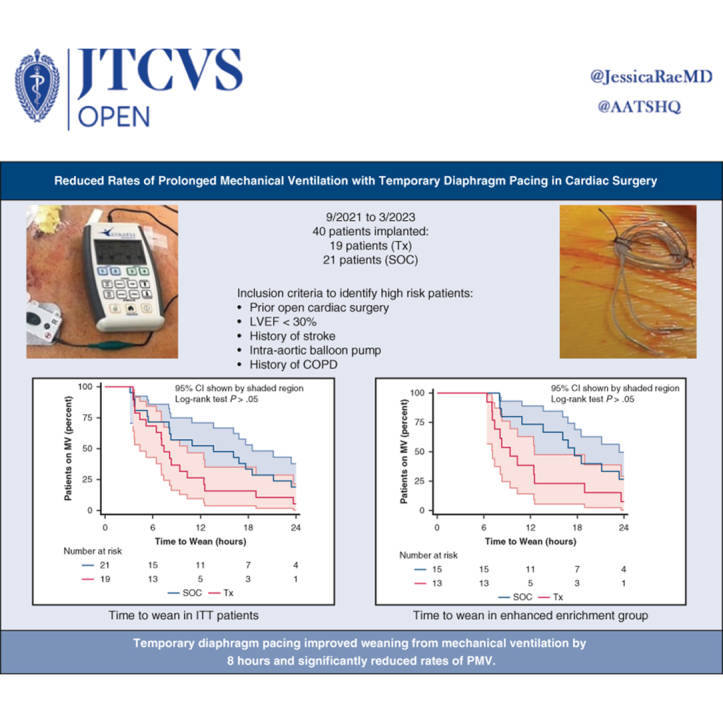

**Figure:**
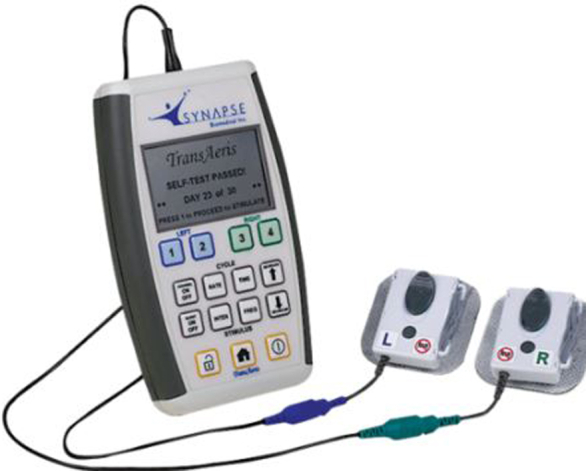
External pulse generator to stimulate the diaphragm.

**Central Message**

Temporary DP improved weaning from MV by 8 hours with a significant reduction of PMV. Multicenter trials confirming DP as an ERAS tool may reduce length of stay, postoperative infections, and costs.

**Perspective**

PMV and VIDD are still significant problems in cardiac surgery, leading to increased morbidity especially in high-risk populations. Temporary DP reduced the relative risk of being on the ventilator by 71% at 24 hours. This research, if confirmed in multicenter trials, will help address this problem and decrease MV time.

Prolonged mechanical ventilation (PMV) after cardiac surgery, which has been defined as mechanical ventilation (MV) more than 24 hours after leaving the operating room, occurs in 15% of patients undergoing cardiac surgery in recent reports.^1^ Although common, PMV is not benign. A large single-institution study found an increase in major infections with significant increases starting at 12 hours of MV and exponentially increasing to 35.7% with PMV more than 48 hours.^2^ A multi-year statewide study showed PMV after cardiac surgery contributed a 156% increase or $41,700 additive cost per patient, which was greater than the added cost per patient for permanent stroke, reoperation for bleeding, or atrial fibrillation.^3^ When the specific number of patients with various complications were taken into account, PMV was the greatest overall contributor to increased costs of all of the complications measured.^3^ Given that PMV is associated with substantial increases in morbidity and healthcare costs, strategies directed toward decreasing its incidence are consequential.

A recent joint consensus statement that included the Enhanced Recovery After Surgery (ERAS) Cardiac Society stated there is moderate quality of evidence that early extubation less than 6 hours will decrease intensive care unit (ICU) and hospital length of stays.^4^ PMV is considered a quality metric because it can lead to higher mortality rates, especially when evaluating early survival, and higher rates of readmission to the ICU.^1^ In a group of 4809 patients, Sankar and colleagues^1^ found that 15% of patients remained on MV at 24 hours, with a median time of 8.9 hours and associated a significant increase in several postoperative complications with MV more than 12 hours.^1^ This study also found that impaired ventricular function and previous cardiac surgery were associated with PMV.^1^

The etiology of PMV is multifactorial, but diaphragm muscle atrophy has been shown to be a significant contributor. Research in animals and humans has shown even short periods of MV leads to biochemical and histopathologic changes manifesting as diaphragm muscle atrophy.^5^ Clinically, this condition is called “ventilator-induced diaphragm dysfunction” (VIDD). Atrophy and VIDD increase in severity with increasing time on MV, but direct stimulation of muscle has been shown to reduce or reverse muscle atrophy.^6^^,^^7^ Even during the operative time of the initial cardiac surgery, changes to the diaphragm occur, and those changes can be overcome with periodic hemidiaphragm stimulation in direct comparison of the 2 hemidiaphragms during the index operation.^6^ VIDD occurs in patients undergoing cardiac surgery, and there is an identified clinical need for treatments to prevent the muscle atrophy that leads to VIDD. Direct stimulation with diaphragm pacing (DP) is efficacious in weaning patients with high spinal cord injuries from MV and providing primary ventilatory support. In patients with acute spinal cord injuries, DP has been shown to reduce postoperative ventilation times by 64%.^8^ There has been growing evidence of the use of temporary phrenic nerve stimulation or DP to prevent VIDD and ventilator-induced lung injury.^9^^,^^10^ The impact DP has on ventilation times after cardiac surgery has not been fully evaluated.

The initial prospective Food and Drug Administration (FDA) feasibility trial of the temporary DP device in 12 patients showed ease of placement, removal, and functionality of the electrodes. Electrode stimulation surpassed ideal tidal volumes, indicating effectiveness in preventing diaphragm atrophy.^11^

An initial pilot study using temporary DP electrodes in patients undergoing cardiac surgery in 32 patients showed it is safe and feasible. There were 10 patients with PMV at 24 hours in this group who then began stimulation with the electrodes. The median percent of time on MV in the first 120 hours postoperatively was 35.7% in the stimulated group versus 80.0% in the nonstimulated group.^12^ There have been several cohort observation reports of immediate use of temporary DP when patients arrive in the ICU in lung transplant recipients (30 subjects) and high-risk patients with vascular disease (14 subjects) with thoracoabdominal incisions.^13^^,^^14^ In both of these reports, there were no adverse events and a positive assessment of improvement of respiratory function.

We hypothesize that immediate DP on admission to the ICU will prevent VIDD and decrease time on MV. DP has been shown to treat VIDD and accelerate MV weaning in other patient groups. This study will analyze the roll of DP in high-risk cardiac surgical patients in a randomized, controlled trial.

## Material and Methods

### Study Design

This study was conducted as a randomized prospectively designed study and was initiated at a single institution, University Hospitals Cleveland Medical Center. The study was registered with clinicaltrials.gov (NCT04899856) and conducted in accordance with the Declaration of Helsinki. The local Institutional Review Board approved the study before the commencement of patient enrollment (STUDY20210729, initial approval July 6, 2021). All patients, or their authorized representatives, provided informed consent at least 1 day before study participation.

### Patient Selection and Management

Patients who were undergoing an open cardiac procedure by median sternotomy were eligible for participation in this study up to a maximum of 40 patients receiving a DP implant at the time of surgery. Inclusion criteria for patients would be to include at least 1 of the following risk factors: preoperative or anticipated intraoperative intra-aortic balloon pump, any prior open cardiac surgery, left ventricular ejection fraction 30% or less, history of transient ischemic attack or cerebrovascular accident, or history of chronic obstructive pulmonary disease. These risk factors were chosen from the initial pilot trial patients who had the greatest likelihood of PMV that involved 32 patients with temporary DP electrode implants and 10 patients who were on MV at 24 hours.^12^ The FDA has recently given guidance on the use of enrichment patient populations in the prospective use of any patient characteristic to select a study population in whom detection of an effect is more likely than it would be in an unselected population.^15^ These enrichment strategies are intended to increase the efficiency of device development and support precision medicine, so that low-risk patients for PMV would not need to be involved in the research protocol. Because the FDA classifies anyone aged 21 years or less as a child, our study enrolled only patients aged more than 22 years. Exclusion criteria included preexisting MV, known phrenic nerve paralysis, and progressive, nonreversible neuromuscular disease affecting the diaphragm. Patients who were pregnant or lactating were also excluded. Patients receiving a left ventricular assist device were not enrolled in this study. Preoperative demographics and relevant medical/surgical history were collected during the screening process. The type of open cardiac surgery the patient underwent was also recorded.

**Figure 1:**
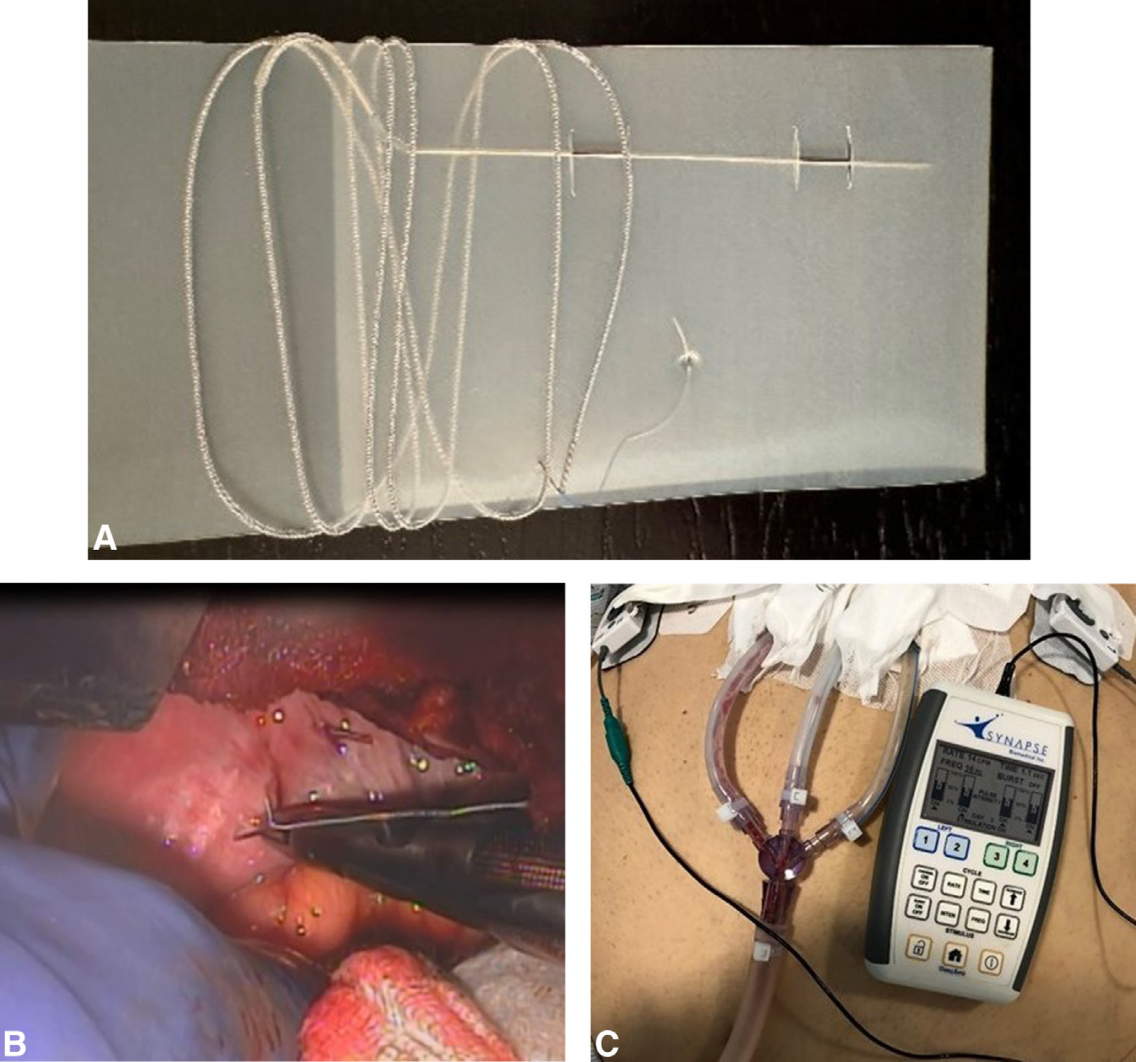
A, Temporary DP electrode. B, Placing the second DP electrode in the left diaphragm in the diaphragm muscle lateral to central tendon near the entry point of phrenic nerve. C, Stimulator attached to percutaneously tunneled wires.

All patients with standard of care (SOC) underwent the same ERAS protocols before surgery intraoperatively and postoperatively to extubate within 6 hours postoperatively. This included rapid reversal of paralytics if needed, nonopioid pain control, and discontinuing sedation. Patients were randomized to treatment (Tx) or control SOC groups after placement and before arrival to the ICU. The DP electrodes were attached to the stimulator, which was turned on arrival to the ICU for patients in the Tx group (Figure 1). Stimulator programming was optimized to elicit diaphragm recruitment without pain or discomfort due to stimulation. Programmable parameters included intensity, frequency, inspiratory time, respiratory rate, and pulse burst on or off. Initial default settings were evaluated for each and adjusted for each patient. The respiratory rate was set to 1 breath per minute higher than the mechanical ventilator setting. This was done so that the stimulation, if strong enough to elicit a diaphragm contraction, would trigger the ventilator and avoid any asynchronies. The stimulus intensity was adjusted upward until the patient reached the threshold for pain or discomfort and then was reduced to below that threshold. Stimulus frequency was increased, if necessary, to provide a tetanic fused contraction or more comfortable contraction. Stimulation was then left on continuously unless an adverse event occurred that necessitated turning stimulation off. Adverse events included electrode-induced arrhythmia, pain, or change in patient status necessitating cessation of stimulation per surgeon evaluation. Throughout the stimulation period, intensity and frequency could be increased or decreased by clinical personnel to elicit a stronger contraction or to address pain/discomfort issues. No patients experienced these adverse events, and stimulation was continued through extubation for all enrolled subjects.

All electrodes were removed without complications at bedside before discharge by gently pulling on the externalized portion of the electrodes before discharge from the ICU or 30 days after implantation, whichever came first. Thirty days after electrode removal, patients were administered a follow-up questionnaire by phone, email, or visit to discuss any readmission or reintubation, postprocedure complications, and their location (eg, home, nursing home, long-term acute care hospital)

### Device Description, Implantation, and Operation

The TransAeris System (Synapse Biomedical) is a temporary percutaneous intramuscular diaphragm stimulator. This device includes 4 TransLoc intramuscular diaphragm electrodes, the TransAeris external stimulator, and the FrictionLoc connectors. The device is connected to the surface of the diaphragm, with 2 electrodes in each hemidiaphragm at the motor points. These are the locations on the diaphragm where the phrenic nerve axons enter and branch into many subsequent axon terminals (Figure 1, *A* and *B*). The proximity of the electrodes to the motor point allows the spread of the electrochemical reaction and propagating action potentials induced by the stimulus charge to recruit the desired contraction strength without the risk of axonal degeneration or demyelination from contact with the phrenic nerve. The level and coordination of stimulus to each electrode are controlled by the external stimulator to provide a smooth and sufficient inspiration (Figure 1, *C*). Electrical activation of the diaphragm through motor point stimulation conditions the muscle to mitigate disuse atrophy.

During implantation, barbs attached to the end of each of the 4 electrodes promote fixation of the device to the diaphragm at the motor points. Adequate electrode length is left to prevent tension during muscle contraction. The electrodes are externalized through the most direct pathway from the thoracic cavity to the skin to facilitate removal.

### Study End Points

The primary safety outcome was incidence of serious device-related adverse events. The primary efficacy end points are time on MV and proportion of patients successfully weaned at 6, 12, 24, 48, and 120 hours postoperatively. Secondary end points reported included hospital and ICU length of stay.

### Data Analysis

This study was designed to gather data on the use of temporary DP in subjects identified before surgery to be at greater risk of PMV after surgery. Patients were considered enrolled if electrodes were successfully implanted and thus included in analysis. Consented patients were excluded if electrodes were not placed, for example, if the surgeon was unable to access the pleural space or an enlarged heart prevented access to left diaphragm. These patients were excluded from randomization. Successful extubation was defined as remaining ventilator free for at least 48 hours. The 2 groups of Tx and SOC were analyzed as intention-to-treat (ITT) comparing both groups. Patients were further analyzed with predictive enrichment to look at the patient group who might best benefit from DP, that is, those who were able to be rapidly extubated.^15^ Patients who were extubated in less than 6 hours were excluded so that a comparison could be made between the Tx and SOC in patients who were extubated after 6 hours. These groups are identified as predictive enrichment intent to treat (pITT). Because the sequela of VIDD is dependent on the time on MV, patients who are on MV longer would more likely respond to DP treatment. Therefore, using predictive enrichment as per FDA guidance, analyzing patients more likely to respond to treatment would include patients on MV greater than 6 hours than those not requiring MV beyond 6 hours.

Continuous data were summarized using descriptive statistics (means with SDs and medians with interquartile ranges [IQRs]), and categorical variables were summarized using frequency counts and percentages. Reported *P* values are 2 sided.

## Results

### Enrollment and Baseline Characteristics

**Figure 2:**
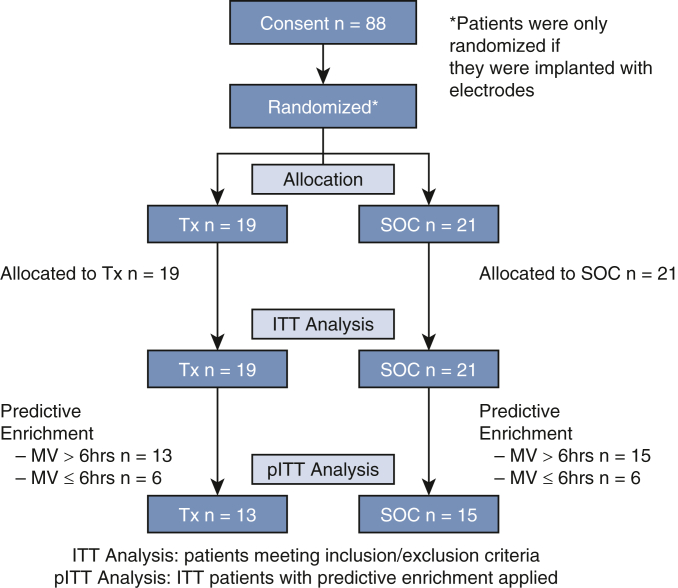
CONSORT diagram. *Tx*, Treatment; *SOC*, standard of care; *MV*, mechanical ventilation.

Of the 88 patients who provided informed consented, 40 received implants between September 2021 and March 2023 with the flow of patients outlined in the CONSORT diagram (Figure 2). The remaining 48 patients did not receive implants for various reasons, including could not access the diaphragm (22), no reason (18), did not enter pleural space (3), patient unstable (3), and changed operation from median sternotomy (2). A total of 21 patients were randomized to SOC and 19 patients to Tx. Table 1 outlines the demographics for these groups including the pITT groups along with the patients who did not receive an implant. There was no statistical differences between any of the compared groups: the SOC versus Tx for all patients; the SOC versus Tx for the pITT groups; for patients on MV less than 6 hours or greater than 6 hours; and for patients receiving or not receiving implants. Table 1 also lists the inclusion criteria of risk factors for PMV that the patients had with no statistical difference between these groups.

**Table 1:** Patient demographics

Variable	ITT			pITT			Implant recipients			Consented		
	SOC	Tx	*P*	SOC	Tx	*P*	MV ≤6 h	MV >6 h	*P*	Implanted	Not implanted	*P*
n	21	19		15	13		12	28		40	48	
Female (%)	7 (33%)	8 (42%)	NS	5 (33%)	7 (54%)	NS	3 (25%)	12 (43%)	NS	15 (38%)	13 (27%)	NS
Age, y	65.6 ± 9.5	65.3 ± 11.1	NS	64.5 ± 10.0	63.0 ± 11.7	NS	69.4 ± 8.2	63.8 ± 10.6	NS	65.5 ± 10.2	63.8 ± 9.6	NS
BMI	30.5 ± 5.8	31.2 ± 9.0	NS	31.1 ± 6.0	29.7 ± 9.0	NS	31.8 ± 7.5	30.4 ± 7.4	NS	30.8 ± 7.4	31.3 ± 6.4	NS
Arrythmia	7 (33%)	6 (32%)	NS	6 (40%)	5 (38%)	NS	2 (17%)	11 (39%)	NS	13 (33%)	17 (36%)	NS
Cardiac pacer/ICD	1 (5%)	2 (11%)	NS	1 (7%)	2 (15%)	NS	0	3 (11%)	NS	3 (8%)	4 (9%)	NS
LVEF	49 ± 17%	48 ± 18%	NS	49 ± 15%	47 ± 18%	NS	49 ± 19%	48 ± 17%	NS	48 ± 17%	47 ± 14%	NS
Diabetes	7 (33%)	6 (32%)	NS	6 (40%)	4 (30%)	NS	3 (25%)	10 (36%)	NS	12 (33%)	20 (43%)	NS
NIV	1 (5%)	3 (16%)	NS	0	2 (15%)	NS	2 (17%)	2 (7%)	NS	4 (10%)	2 (4%)	NS
Hypertension	16 (76%)	15 (79%)	NS	12 (80%)	10 (77%)	NS	9 (75%)	22 (79%)	NS	31 (78%)	31 (66%)	NS
PVD	1 (5%)	1 (5%)	NS	0	1 (8%)	NS	1 (8%)	1 (4%)	NS	2 (5%)	4 (9%)	NS
Renal failure	4 (19%)	4 (21%)	NS	3 (20%)	2 (15%)	NS	3 (25%)	5 (18%)	NS	8 (20%)	7 (15%)	NS
PMV risk												
Prior cardiac surgery	6 (29%)	7 (37%)	NS	4 (27%)	5 (38%)	NS	9 (32%)	7 (24%)	NS	16 (40%)	19 (40%)	NS
LVEF ≤30%	4 (19%)	6 (32%)	NS	2 (13%)	4 (31%)	NS	8 (29%)	6 (21%)	NS	14 (35%)	6 (13%)	NS
History of TIA or CVA	3 (14%)	4 (21%)	NS	2 (13%)	3 (23%)	NS	9 (32%)	4 (14%)	NS	13 (33%)	18 (38%)	NS
IABP	4 (19%)	2 (11%)	NS	4 (27%)	2 (15%)	NS	1 (4%)	7 (24%)	NS	8 (20%)	4 (8%)	NS
COPD	7 (33%)	6 (32%)	NS	6 (40%)	4 (31%)	NS	6 (21%)	10 (34%)	NS	16 (40%)	19 (40%)	NS

ITT analysis: patients meeting inclusion/exclusion criteria. pITT analysis: ITT patients with predictive enrichment applied in those on mechanical ventilator more than 6 hours. *ITT*, Intention-to-treat; *pITT*, predictive enrichment intent to treat; *SOC*, standard of care; *Tx*, treatment; *MV*, mechanical ventilation; *NS*, not significant; *BMI*, body mass index; *ICD*, implantable cardioverter defibrillator; *LVEF*, left ventricular ejection fraction; *NIV*, non-invasive ventilation; *PVD*, peripheral vascular disease; *PMV*, prolonged mechanical ventilation; *TIA*, transient ischemic attack; *CVA*, cerebrovascular accident; *IABP*, intra-aortic balloon pump; *COPD*, chronic obstructive pulmonary disease.

Table 2 lists the procedures that the patients underwent along with the cardiopulmonary bypass time and operating room time for each of these groups of patients. The most common other procedure involved the addition of a tricuspid valve procedure in 6 subjects. There were 2 fewer patients in the no implant groups because the operation did not involve a median sternotomy. There were no significant differences in any of the groups in cardiopulmonary bypass time or operative time in any of the subgroups that were analyzed.

**Table 2:** Procedure performed and operative factors

Variable	ITT			pITT			ITT			Consented		
	SOC	Tx (Stim)	*P*	SOC	Tx (Stim)	*P*	MV ≤6 h	MV >6 h	*P*	Implanted	Not implanted	*P*
n	21	19		15	13		12	28		40	46	
Procedure type												
Isolated CABG	5 (24%)	6 (32%)		3 (18%)	4 (33%)		13 (46%)	7 (24%)		11 (28%)	8 (17%)	
Isolated AVR											4 (9%)	
Isolated MVR	1 (5%)	1 (5%)								2 (5%)		
AVR + CABG	3 (14%)			2 (12%)			1 (4%)	2 (7%)		3 (8%)	2 (4%)	
MVR + CABG											1 (2%)	
MV repair		1 (5%)			1 (8%)			1 (3%)		1 (3%)	2 (4%)	
MV repair + CABG							1 (4%)					
Other	12 (57%)	11 (58%)		12 (71%)	7 (58%)		13 (46%)	19 (66%)		23 (58%)	29 (63%)	
CPB time (min)	125 ± 49	112 ± 31	NS	135 ± 52	116 ± 32	NS	100 ± 25	126 ± 44	NS	119 ± 41	128 ± 60	NS
OR time (h)	6.3 ± 1.3	6.3 ± 0.9	NS	6.5 ± 1.4	6.4 ± 1.0	NS	6.1 ± 1.0	6.4 ± 1.2	NS	6.3 ± 1.1	6.7 ± 1.7	NS

ITT analysis: patients meeting inclusion/exclusion criteria. pITT analysis: ITT patients with predictive enrichment applied. *ITT*, Intention-to-treat; *pITT*, predictive enrichment intent to treat; *SOC*, standard of care; *Tx*, treatment; *MV*, mitral valve; *CABG*, coronary artery bypass graft; *AVR*, aortic valve replacement; *MVR*, mitral valve replacement; *CPB*, cardiopulmonary bypass; *NS*, not significant; *OR*, operating room.

### Safety Analysis

Device-related adverse effects were monitored during the trial. These effects included failure of electrodes to capture, occurrence of device-induced arrhythmia, and inability to remove electrodes. There were no device-related serious adverse device effects and no adverse device effects. One electrode disconnected from FrictionLoc and 1 broken FrictionLoc were identified and corrected with no effect. There were no interactions with stimulation and any of the care in the ICU. All implanted electrodes were safely removed at the end of use.

### End Point Evaluation

**Figure 3:**
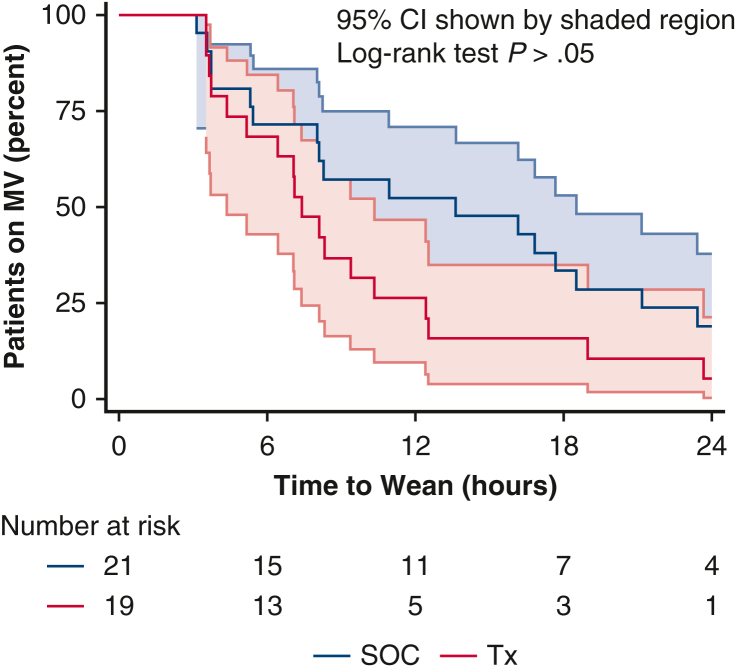
Proportion of MV time: SOC versus diaphragm stimulation therapy in implant recipients. *MV*, Mechanical ventilation; *SOC*, standard of care; *Tx,* treatment.

The Tx group included 19 patients, and the SOC group included 21 patients. The Tx group received TransAeris stimulation upon entry to the ICU and the SOC group did not. Table 3 summarizes the primary efficacy outcomes of time on MV. In the ITT SOC population, the time on MV averaged 13.6 hours (range, 5.4-18.5), whereas in the Tx group it was 7.4 hours (range, 4.4-10.3), which did not reach statistical significance. Although the proportion of time on MV in the ITT group also trended toward a positive effect of Tx, it did not reach significance. The ICU length of stay and hospital length of stay were not significantly different. The analysis of the ITT population yields the time to event curve shown in Figure 3. It can be seen that 52% of the SOC group and 26% of the Tx group were still on MV at 12 hours.

**Table 3:** Efficacy outcomes of implant recipients: Time on mechanical ventilation

Variable	ITT		pITT	
	SOC	Tx (Stim)	SOC	Tx
n	21	19	15	13
Proportion weaned				
≤6 h	6 (29%)	6 (32%)	–	–
6-12 h	4 (19%)	8 (42%)	4 (27%)	8 (62%)
12-24 h	7 (33%)	4 (21%)	7 (47%)	4 (31%)
24-48 h	2 (10%)	1 (5%)	2 (13%)	1 (8%)
>48 h	2 (10%)	0	2 (13%)	0
MV time (h)	13.6 [5.4-18.5]	7.4 [4.4-10.3]	17.7 [8.3-23.4]∗	9.4 [7.1-12.5]∗
Proportion of time on MV (median, IQR)				
First 12 h on MV (%)	100 [45.3-100]	61.7 [36.4-100]	100 [91.0-100]∗	78.1 [59.2-100]∗
First 24 h on MV (%)	56.8 [22.6-88.1]	30.8 [18.2-51.7]	73.6 [45.5-100.0]∗	39.0 [29.6-79.0]∗
First 48 h on MV (%)	28.4 [11.3-44.1]	15.4 [9.1-25.9]	36.8 [22.7-59.4]∗	19.5 [14.7-39.5]∗
First 120 h on MV (%)	11.4 [4.5-17.6]	6.2 [3.6-10.3]	14.7 [9.1-23.8]∗	7.8 [5.9-15.8]∗
ICU length of stay (h)	149 [53-186]	123 [46-174]	166 [101-203]	152 [117-190]
Hospital length of stay (d)	12 [9-21]	14 [9-24]	14 [9-31]	14 [9-24]

*ITT*, Intention-to-treat; *pITT*, predictive enrichment intent to treat; *SOC*, standard of care; *Tx*, treatment; *MV*, mechanical ventilation; *IQR*, interquartile range; *ICU*, intensive care unit.

∗ Statistically significant difference between SOC and Tx (*P* < .05).

**Figure 4:**
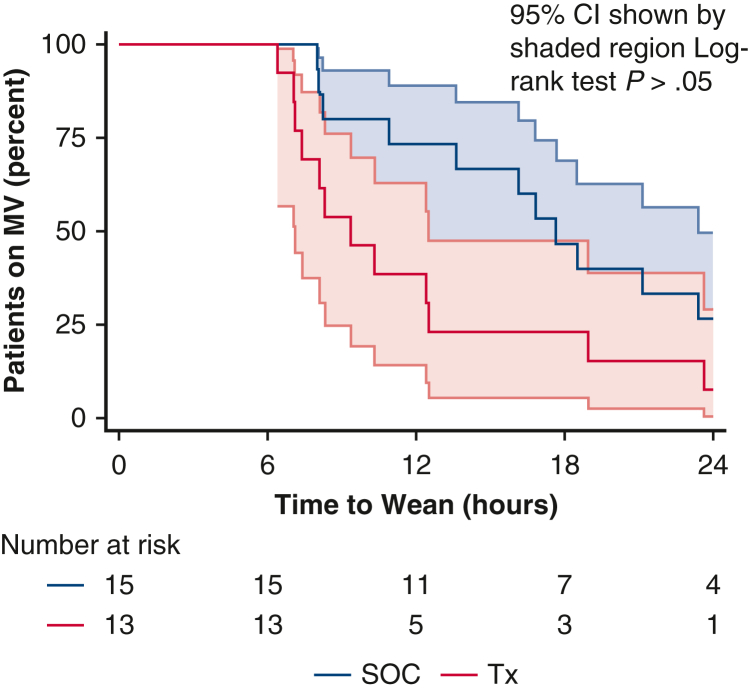
Proportion of MV time in enriched patients: SOC versus diaphragm stimulation therapy. *MV*, Mechanical ventilation; *SOC*, standard of care; *Tx*, treatment.

Overall, 28 of the 40 patients required MV beyond 6 hours postoperatively. There were 15 of the 21 patients in the SOC group (71.4%) and 13 of 19 patients in the Tx group (68.4%) who required MV beyond 6 hours, which became the pITT cohort. There was a higher percentage of patients on MV beyond 6 hours than expected in cardiac surgery because of the use of the prognostic enrichment of the patient population. In the pITT SOC, the median time on MV was 17.7 hours (IQR, 8.3-23.4), whereas in the pITT Tx group this was 9.4 hours (IQR, 7.1-12.5), which was 8 less hours of MV and reached statistical significance (*P* < .05). The proportion of time on MV also reached significance for MV at 12 hours, 24 hours, 48 hours, and 120 hours. There was no significant difference in ICU stay or hospital length of stay. Figure 4 highlights this enhanced enrichment group on weaning in the first 24 hours.

Focusing on the pITT analysis population intended to benefit from the treatment (ie, those patients on MV for >6 hours) yields the time to event analysis shown in Table 4, which outlines the relative risk reduction of using DP immediately on entry to the ICU. This shows that 73% of the SOC group were still on MV at 12 hours with a 17.7-hour (95% CI, 8.3-23.4) median time to wean compared with 38% of the Tx patients still on MV at 12 hours with a 9.4-hour (95% CI, 7.1-12.5) median time to wean. This translates to a 47.5% relative risk reduction with use of DP. At 24 hours, 27% of SOC patients were on MV, whereas only 8% of the Tx group were on MV. Patients on MV between 6 and 24 hours are the primary population of interest in receiving potential benefit from DP treatment.

**Table 4:** Relative risk reduction in mechanical ventilation duration using diaphragm pacing in patients ventilated for more than 6 hours (total patients in this group n = 28)

MV duration	SOC		Tx		RRR
MV >6 h	15		13		
MV >12 h	11	73.3%	5	38.5%	47.5%
MV >24 h	4	26.7%	1	7.7%	71.2%
MV >48 h	2	13.3%	-	0.0%	100.0%
MV >120 h	2	13.3%	-	0.0%	100.0%

RRR = (SOC%-Tx%)/SOC%. *SOC*, Standard of care; *Tx*, treatment; *RRR*, relative risk reduction; *MV*, mechanical ventilation.

## Discussion

Failure to wean from MV is a major health problem with more than 100,000 tracheostomies performed in the United States for all ICU patients. The COVID-19 pandemic highlighted the shortage of ICU beds for patients. There has been significant research in overcoming VIDD and the atrophy of the diaphragm muscles to help with this healthcare problem with the use of both temporary phrenic and DP technology. Sankar and colleagues^1^ describe PMV in cardiac surgery as leading to increased morbidity and lower early survival. The authors thought there is a lack of adequate perioperative management strategies that improve outcomes for cardiac surgical patients requiring PMV. This report is the first randomized trial to address this problem in cardiac surgery.

The recommended guideline for ventilator extubation after open cardiac procedures is to wean by 6 hours postoperatively. The Society of Thoracic Surgeons performance measures include a quality performance measure for PMV, defined as the need for intubation more than 24 hours. Temporary DP is a novel approach to reduce PMV and its associated comorbidities that can be placed at the time of the index operation. In both a pilot study and a randomized controlled trial, it has demonstrated efficacy. This study found that DP upon arrival to the ICU decreased the median time on MV by 8 hours in a high-risk enriched patient population. DP placement and use are safe, as evidenced by the lack of adverse device- and procedure-related events.

This is a critical area of care where DP can positively impact these patients’ short- and long-term outcomes. Muscle fibers of the diaphragm become atrophic the longer a patient requires ventilatory support. The severity of atrophy increases with time, and cardiac surgery dysfunction occurs during the operation; intraoperative stimulation has even been shown to help.^6^ Early intervention with electrical stimulation can reduce this atrophy and its associated clinical effects. Even when analyzing all patients, the Tx group had a 71% relative risk reduction of requiring MV at 24 hours postoperatively, which if confirmed in a multicenter trial would decrease the known morbidity and mortality associated with being on MV at 24 hours in patients undergoing cardiac surgery.

### Limitations

This study has limitations inherent to a single-site study with historical experience in DP, which can limit generalizability. Part of this study was done during some peaks of COVID-19 hospitalizations that affected the analysis of ICU and hospital length of stay limiting analysis. Also, patients who may have benefited from being included, such as in emergency high-risk surgeries, were excluded because of Institutional Review Board limitations of consenting at least 24 hours before surgery. Patients who consented but did not receive an implant were not analyzed, but there did not seem to be any significant demographic or operative differences. Future randomized trials can overcome these limitations.

## Conclusions

Temporary DP is safe and easily used by surgeons, and did not interfere with ICU care. Preliminary results show a decrease in PMV as diaphragm stimulation improves ventilator weaning, thus decreasing associated comorbidities and healthcare costs. The conclusions from the randomized investigation are that a pivotal multicenter trial is needed to establish risk-prediction models for patients requiring PMV and to establish ERAS protocols addressing diaphragm dysfunction for patients undergoing open cardiac surgery.

### Webcast

You can watch a Webcast of this AATS meeting presentation by going to: https://www.aats.org/resources/randomized-study-of-temporary-7360.

**Figure:**
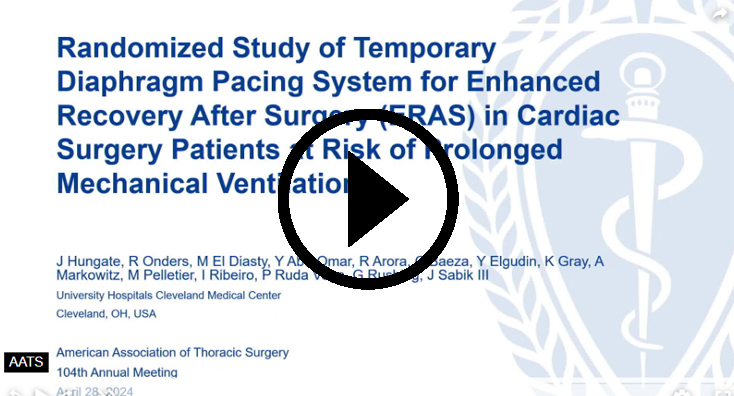

## Conflict of Interest Statement

Dr Onders, University Hospitals Cleveland Medical Center, and Case Western Reserve University have intellectual property rights and equity in Synapse Biomedical, which manufactures the device used in the study. Dr Onders is also a consultant, board member, and Chief Medical Officer of Synapse Biomedical. All other authors reported no conflicts of interest.

The *Journal* policy requires editors and reviewers to disclose conflicts of interest and to decline handling or reviewing manuscripts for which they may have a conflict of interest. The editors and reviewers of this article have no conflicts of interest.
